# The ability to manipulate plant glucosinolates and nutrients explains the better performance of *Bemisia tabaci* Middle East‐Asia Minor 1 than Mediterranean on cabbage plants

**DOI:** 10.1002/ece3.2921

**Published:** 2017-06-30

**Authors:** Hongying Cui, Litao Guo, Shaoli Wang, Wen Xie, Xiaoguo Jiao, Qingjun Wu, Youjun Zhang

**Affiliations:** ^1^ Department of Plant Protection Institute of Vegetables and Flowers Chinese Academy of Agricultural Sciences Beijing China; ^2^ College of Life Science Hubei University Wuhan China

**Keywords:** *Bemisia tabaci*, cabbage, free amino acids, glucosinolate, MEAM1 and MED

## Abstract

The performance of herbivorous insects is greatly affected by host chemical defenses and nutritional quality. Some herbivores have developed the ability to manipulate plant defenses via signaling pathways. It is currently unclear, however, whether a herbivore can benefit by simultaneously reducing plant defenses and enhancing plant nutritional quality. Here, we show that the better performance of the whitefly *Bemisia tabaci* Middle East‐Asia Minor 1 (MEAM1; formerly the “B” biotype) than Mediterranean (MED; formerly the “Q” biotype) on cabbage is associated with a suppression of glucosinolate (GS) content and an increase in amino acid supply in MEAM1‐infested cabbage compared with MED‐infested cabbage. MEAM1 had higher survival, higher fecundity, higher intrinsic rate of increase (*r*_m_), a longer life span, and a shorter developmental time than MED on cabbage plants. Amino acid content was higher in cabbage infested with MEAM1 than MED. Although infestation by either biotype decreased the levels of total GS, aliphatic GS, glucoiberin, sinigrin, glucobrassicin, and 4OH‐glucobrassicin, and the expression of related genes in cabbage, MED infestation increased the levels of 4ME‐glucobrassicin, neoglucobrassicin, progoitrin, and glucoraphanin. The GS content and expression of GS‐related genes were higher in cabbage infested with MED than with MEAM1. Our results suggest that MEAM1 performs better than MED on cabbage by manipulating host defenses and nutritional quality.

## 1 INTRODUCTION

When attacked by herbivorous insects, many plants respond by increasing their secondary metabolites that are toxic to insects (Elbaz et al., 2012; Mewis et al., 2006; Wei et al., 2011). Glucosinolates (GS), which are found exclusively in the Brassicaceae and related families, are represented by compounds that have approximately 100 different side chains bonded to a core molecule containing sulfur and nitrogen (Halkier & Gershenzon, 2006). Based on the side chain structure, GS are typically divided into three classes: aliphatic GS derived principally from Ala (alanine), Val (valine), Leu (leucine), Ile (isoleucine), and Met (methionine); indolic GS derived from Trp (tryptophan); and aromatic GS derived from Phe (phenylalanine) and Tyr (tyrosine) (Markovich et al., 2013; Santolamazza‐Carbone, Velasco, Soengas, & Cartea, 2014; Textor & Gershenzon, 2009). In addition to depending on primary metabolism for the synthesis of amino acids, GS biosynthesis depends on secondary metabolism pathway enzymes (Kempema, Cui, Holzer, & Walling, 2007). GS can be cleaved and activated by the enzyme myrosinase (β‐thioglucoside glucohydrolase) upon tissue damage, leading to the production of a variety of toxic degradation products, including isothiocyanates, nitriles, thiocyanates, oxazolidine‐2‐thiones, and epithionitriles (Elbaz et al., 2012; Hopkins, van Dam, & van loon, 2009; Müller & Sieling, 2006). GS negatively affect a wide range of herbivorous insects, for example, they can deter feeding and inhibit growth and can also be acutely toxic, although the specialist herbivore, *Pieris rapae*, is not affected by GS (Halkier & Gershenzon, 2006; Renwick & Lopez, 1999).

Regulation of GS biosynthesis involves enzymes belonging to the cytochrome P‐450 monooxygenase gene family (Kempema et al., 2007; Mewis et al., 2006). Expression of genes related to the biosynthesis of aliphatic GS (*CYP79F1* and *CYP83A1*) and of indolic GS (*CYP79B2* and *CYP83B1*) could affect the performance of insects (Gols, Wagenaar et al., 2008; Newton, Bullock, & Hodgson, 2009). It remains unclear, however, whether the GS pathway affects the performance of phloem‐feeding insects such as the whiteflies that feed on *Brassica oleracea*.

In addition to being affected by secondary metabolites, host quality for herbivores is also affected by nutrient content. Host proteins, free amino acids, and carbohydrates affect host acceptance by herbivores (Gols, Bukovinszky et al., 2008). Researchers have used amino acid composition to compare host plant suitability among plant species or cultivars (Chiozza, Oneal, & Macintosh, 2010; Wilkinson & Douglas, 2003). For example, His (histidine), Pro (proline), Asn (asparagine), Glu (glutamic acid), Gln (glutamine), and Ser (serine) may be associated with nutritional quality and could be important in determining host suitability (Karley, Douglas, & Parker, 2002). In general, 10 amino acids are considered to be essential for insects: Arg (arginine), His, Ile, Leu, Lysine (lysine), Met, Phe, Thr (threonine), Trp, and Val (Louis, Singh, & Shah, 2012).

By altering source‐sink relationships, insect feeding can influence plant nutrient availability and thus influence insect performance (Leroy et al., 2011; Petersen & Sandstrom, 2001). For example, infestation by *Bemisia tabaci* Middle East‐Asia Minor 1 (MEAM1) (formerly called the “B” biotype) induced decreases in amino acids content of leaf, which reduced the fitness of whiteflies that subsequently fed on the leaves (Cui, Sun, Su, Li, & Ge, 2012). Because free amino acids are involved in secondary plant metabolism and in the biosynthesis of GS, phenolics, and other compounds that directly or indirectly affect plant–environment interactions, researchers have determined free amino acid profiles in *Brassica* species (Gomes & Rosa, 2001; Oliveira et al., 2008). The changes in plant biochemistry resulting from insect infestation can affect the subsequent settling, feeding, growth, development, and fecundity of herbivorous insects (Bi, Toscano, & Madore, 2003; Ohgushi, 2005).

While plants have evolved mechanisms to reduce herbivory, herbivorous insects have evolved ways to manipulate plant defenses for their own benefit (Sarmento et al., 2011). Phloem‐feeding insects, for example, can induce cell wall modifications, reduce photosynthetic activity, manipulate source‐sink relations, and modify secondary metabolism in their hosts to their own benefit (Thompson & Goggin, 2006; Walling, 2008). Additional evidence for herbivore manipulation of plant defenses for herbivore benefit is accumulating from studies with phloem‐feeding insects (Pieterse & Dicke, 2007; Thompson & Goggin, 2006). For example, aphid secretes watery saliva containing proteins that are able to bind calcium and prevent calcium‐induced sieve element (SE) occlusion into their feeding sites to suppress the local defense responses (Will & van Bel, 2008). Aphids are able to manipulate the levels of amino acids they ingest so as to improve diet quality (Dardeau et al., 2015; Girousse, Moulia, Silk, & Bonnemain, 2005). Previous studies have not determined, however, whether herbivorous insects can benefit by simultaneously modifying both host defenses and nutrients.

*Bemisia tabaci* (Gennadius) (Homoptera: Aleyrodidae; Gennadius, 1889) is an invasive and damaging pest of field and greenhouse crops worldwide (Bird & Kruger, 2006). It has a broad host plant range that includes several Brassicaceae crops such as cabbage, cauliflower, and broccoli (McKenzie, Hodges, Osborne, Byrne, & Shatters, 2009). *B. tabaci* is recognized as a species complex that comprises a large number of genetically distinct sibling species and/or biotypes (De Barro, Liu, Boykin, & Dinsdale, 2011; Dinsdale, Cook, Riginos, Buckley, & De Barro, 2010). These biotypes differ genetically as well as in host range, host plant adaptability, fecundity, insecticide resistance, capacity to cause plant disorders, and ability to transmit viruses (Chiel et al., 2007; Jones, 2003). MEAM1 and Mediterranean (MED; formerly the “Q” biotype) are regarded as the two most invasive and widely distributed biotypes (Pan et al., 2013, 2015).

Although both biotypes have wide host ranges, MEAM1 and MED differ in host suitability (Iida, Kitamura, & Honda, 2009; Jiao et al., 2013). In our previous field investigations in China, we found that cabbage was frequently infested with MEAM1 but seldom with MED (Pan et al., 2015). The objectives of this study were to compare the performance of MEAM1 and MED on cabbage plants and then to determine the reasons for the differences in performance.

## 2 MATERIALS AND METHODS

### 2.1 Insect source and host plants

Specimens of *B. tabaci* MEAM1 were originally collected from cabbage (*Brassica oleracea* var. Jingfeng1) in 2004 in Beijing. Specimens of *B. tabaci* MED were originally collected from poinsettia (*Euphorbia pulcherrima*) in 2008 in Beijing. To minimize any effects of the source plants on the experiments, we maintained the two biotypes of *B. tabaci* on cotton (a highly suitable host for both species) in separate insect‐proof screened cages (60 × 60 × 60 cm) for at least 10 generations at 25 ± 1°C, 60% ± 10% RH, and 14L: 10D before the experiments were conducted (Jiao et al., 2013). The purity of the two cultures was monitored every generation based on the haplotypes of the DNA sequence obtained using the mtCOI primers (Zhang et al., 2005). Cabbage plants (Jingfeng1) were grown in a potting mixture of peat moss, vermiculite, organic fertilizer, and perlite (10:10:10:1 by volume) in a greenhouse under natural light at 26 ± 2°C. The cabbage plants used for the experiments were established in 12‐cm‐diameter plastic pots (one plant per pot). Plants were used for experiment when they had five to six leaves, at which time they were about 40 days old.

### 2.2 Performance of *B. tabaci* on cabbage

#### 2.2.1 Developmental time, fecundity, and adult longevity

Developmental time, fecundity, and adult longevity for MEAM1 and MED on cabbage were determined in climate chambers (27 ± 0.5°C, 60% ± 10% RH, and 14L: 10D). Cabbage plants of uniform size were randomly selected, and 10 pairs of whitefly adults were allowed to oviposit on each of three leaves per plant; the adults on each leaf were confined in clip cage (circular, 3.5 cm diameter, 1.5 cm height) for 24 hr. After the adults were removed, 10 eggs were retained per leaf (30 eggs per plant), and the leaves were once again enclosed in clip cages. Each biotype was represented by four replicate plants. The four plants (each with 10 eggs × 3 leaves) per biotype were examined daily with a microscope to determine developmental status of the offspring until adult eclosion. After adult eclosion, 32 pairs of newly eclosed adults of each biotype were transferred to another leaf of the same cabbage plant; the leaves were enclosed in clip cages. As before, each biotype was represented by four replicate plants. If a male died, another healthy male from the same treatment was immediately added. Adult longevity and fecundity for each individual whitefly were recorded daily. These procedures and observations were repeated for four generations of *B. tabaci*. New plants (four per biotype) were used for each generation. The data obtained are shown in Fig. S1.

#### 2.2.2 Survival

Survival for MEAM1 and MED on cabbage was determined in climate chambers as described in the previous section. As before, cabbage plants of uniform size were randomly selected, and 10 pairs of whitefly adults were allowed to oviposit on each of three leaves (one leaf per clip cage) per plant. Each biotype was represented by four replicate plants (three leaves per plant, 100–120 eggs per leaves). The adults were removed after 24 hr, and the eggs were allowed to develop. The total number of progeny and their developmental stages (unhatched eggs, first nymphs through pupae, empty exuvia indicating emergence) were recorded. Survival was expressed as the proportion of eggs that developed into adult whiteflies. These procedures and observations were repeated for four generations of *B. tabaci*. New plants (four per biotype) were used for each generation. The data obtained are shown in Fig. S1.

### 2.3 Herbivory treatment

Individuals of *B. tabaci* MEAM1 and MED were collected from the cotton plants that had supported at least 10 generations of the corresponding biotype at 25 ± 1°C, 60% ± 10% RH, and 14L: 10D. Cabbage plants of uniform size were selected as described in the previous sections, and each of three leaves of each plant was infested with 150 MEAM1 or MED whitefly adults (450 whiteflies per plant); each leaf was enclosed in a clip cage. As a control, additional cabbage plants were treated in the same way but were not infested with whiteflies. The plants were kept in climate chambers at 27 ± 0.5°C, 60% ± 10% RH, and 14L: 10D. After 15 days, the infested leaves with the similar densities of MEAM1 and MED were harvested (the data shown in Table S1), and the whiteflies were removed; the noninfested leaves of the control treatment (in the same position on the plant as the infested leaves) were also harvested at this time. The leaves were immediately frozen in liquid nitrogen and transferred to −78°C until they were assayed as described in the following sections. Each treatment (infested with MEAM1, infested with MED, or noninfested control) in each assay was represented by four replicate plants, with three leaves combined and assayed per replicate.

### 2.4 Amino acid determination

Free amino acids were extracted and quantified from the harvested leaves according to Su, Perisser et al. (2015). Amino acids were analyzed by reverse‐phase HPLC with precolumn derivatization using ophthaldialdehyde and 9‐fluorenylmethyloxycarbonyl. Amino acids were quantified using the AA‐S‐17 (PN: 5061‐3331; Agilent Technologies, Palo Alto, CA, USA) reference amino acid mixture, supplemented with asparagine, glutamine, and tryptophan (SigmaAldrich Co., St. Louis, MO, USA). Analyses were performed using an Agilent 1100 HPLC; a reverse‐phase Agilent Zorbax Eclipse C18 column AAA (5 mm, 250 by 4.6 mm) and fluorescence detector were used for chromatographic separation. Amino acids were quantified by comparing peak areas to the standard curve of each reference amino acid. Peak areas were converted to milligram amounts relative to the known internal standard added to each sample; the milligram amounts were corrected for leaf tissue weight.

### 2.5 Glucosinolate analysis

GS in the harvested leaves were analyzed as described by Martinez‐Villaluenga et al. (2009). Briefly, duplicate 200‐mg samples of freeze‐dried leaves were extracted three times with boiling 70% methanol. Glucotropaeolin was added to each sample just before the first extraction as an internal standard for high‐performance liquid chromatography (HPLC) analysis. The isolation, desulfatation, and HPLC analysis of GS were carried out as described by Heaney, Spinks, Hanley, and Fenwick (1986) with modification. Desulfo‐GLS were separated in the Agilent 1100 series HPLC system with an autoinjector (20 μl loop), a Spherisorb ODS‐2 3 Micron column (150 × 4.6 mm), and 1.2 ml/min flow rate at 32°C by eluting with a gradient of water (A) and 20% acetonitrile (B) as follows: isocratically 1% B for 1 min; linear gradient to 99% B for 30 min; isocratically 99% B for 6 min; linear gradient to 1% B for 5 min; and 1% B for 8 min. Desulfo‐GS were detected at λ = 229 nm. The sample content of different types of GS was quantified on the basis of the internal standard.

### 2.6 DNA and RNA extraction and quantitative real‐time PCR (qPCR)

Cabbage leaf tissues were ground in liquid nitrogen. Each treatment combination was repeated for four biological replicates, and each biological replicate contained three technical replicates. Total RNA was extracted with an RNeasy plus Mini kit (Qiagen) following the manufacturer's protocol and was quantified using NanoDrop (Thermo Scientific). After RNA extraction, 1 μg of RNA was used to synthesize the first‐strand cDNA using the PrimeScript RT reagent Kit (TaKaRa) with gDNA Eraser according to the manufacturer's protocol (Su, Oliver et al., 2015).

Real‐time quantitative PCR (qPCR) was used to analyze differences in expression. Gene‐specific primers for the six genes (*CYP79B2*,* CYP83B1*,* CYP79F1*,* CYP83A1*,* Actin*, and *GADPH*) were designed and used in PCR reactions (25 μl) containing 9.5 μl of ddH_2_O, 12.5 μl of 2× SuperReal PreMix Plus (TIANGEN, Beijing, China), 7.5 μmol/L of each specific primer, 1 μl of first‐strand cDNA template, and 0.5 μl of 50× ROX Reference Dye (TIANGEN). Specific primers for the target genes were designed from the cabbage EST sequences using PRIMER5 software. Primer pairs for qRT‐PCR are listed in Table S2. The qPCR program included an initial denaturation for 15 min at 95°C followed by 40 cycles of denaturation for 15 s at 95°C, annealing for 30 s at 60°C, and extension for 32 s at 72°C. For melting curve analysis, an automatic dissociation step cycle was added. Reactions were performed in an ABI 7500 Real‐Time PCR system (Applied Biosystems, USA) with data collection at stage 2, step 3 in each cycle of the PCR reaction. Amplification efficiencies and linear correlation coefficients between the quantity of cDNA template and the quantity of PCR product for the gene‐specific primers were calculated from the dissociation curve of four replicates using five twofold serial dilutions (1:1, 1:2, 1:4, 1:8, and 1:16). Only results with single peaks in melting curve analyses, 95%–100% primer amplification efficiencies, and >.95 correlation coefficients were used for subsequent data analysis. Relative quantification was performed using the 2^−∆∆Ct^ method (Guo et al., 2015; Livak & Schmittgen, 2001). The relative level of the target gene expression was standardized by comparing the copy numbers of target mRNA with copy numbers of Actin and GADPH (the “housekeeping genes”), which remain constant under different treatment conditions. The Actin and GADPH mRNAs of the control were examined in every PCR plate to eliminate any systematic error.

### 2.7 Statistical analyses

Two‐way factorial ANOVAs were used to determine the effects of whitefly biotypes and whitefly generation on the developmental time (from egg to adult), survival, fecundity, life span, and intrinsic rate of increase (*r*_m_) (Table S3, Fig. S1). Calculation of *r*_m_ was based on the age‐stage, two‐sex life table model developed by Chi and Liu (1985) and Chi (1988). One‐way ANOVAs were used to analyze the effects of whitefly species on the following: developmental time, survival, fecundity, life span, and *r*_m_ for the 1st whitefly generation; and properties of cabbage leaves (free amino acid content, GS content, and relative expression of target mRNA [*CYP79B2*,* CYP83B1*,* CYP79F1*,* CYP83A1*]). Cabbage plant was considered a random effect. Differences among means were determined using Tukey's test at *p* < .05. SPSS 13.0 (SPSS Inc., Chicago, IL, USA) was used for statistical analyses.

## 3 RESULTS

### 3.1 Performance of *B. tabaci* MEAM1 and MED on cabbage plants

**Figure 1:**
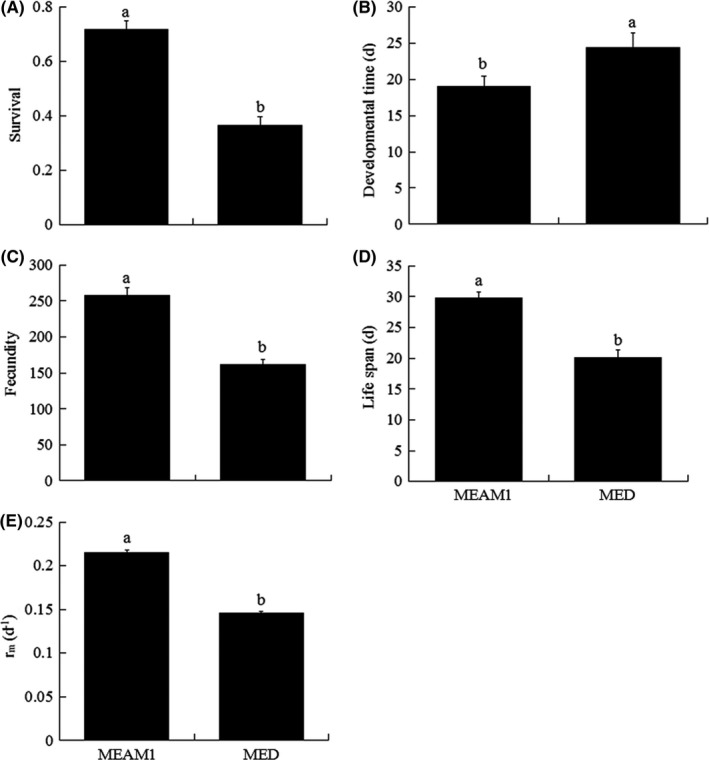
Survival (A), developmental time (from egg to adult) (B), fecundity (C), life span (D), and intrinsic rate of increase (*r*_m_) (E) of the first generation of *B. tabaci* MEAM1 and MED reared on cabbage. Values are means ± *SE*. In each panel, means with different letters are significantly different (*p* < .05)

Survival, developmental time, fecundity, life span, and *r*_m_ on cabbage significantly differed between MEAM1 and MED. Relative to MED, MEAM1 had 96.13% higher survival (*F*_1, 6_ = 149.19, *p* < .0001), 59.76% higher fecundity (*F*_1, 6_ = 33.29, *p* < .001), 27.99% higher *r*_m_ (*F*_1, 6_ = 234.12, *p* < .0001), 48.41% longer life span (*F*_1, 6_ = 46.91, *p* < .0001), and 22.18% shorter developmental time (*F*_1, 6_ = 169.86, *p* < .0001; Figure 1A–E).

The whitefly biotype significantly influenced survival, fecundity, *r*_m_, life span, and developmental time, while whitefly generation significantly influenced fecundity, *r*_m_ and developmental time (Table S3). The interaction between these two factors significantly influenced survival, fecundity and *r*_m_ (Table S3). Relative to MED, MEAM1 had higher survival, fecundity and *r*_m_, longer life span, and shorter developmental time from the 1st to the 4th generation (Fig. S1A–E). The fitness of *B. tabaci* MED was decreased from the 1st to the 4th generation, while *B. tabaci* MEAM1 was unchanged (Fig. S1E).

### 3.2 Free amino acid content of cabbage plants

**Figure 2:**
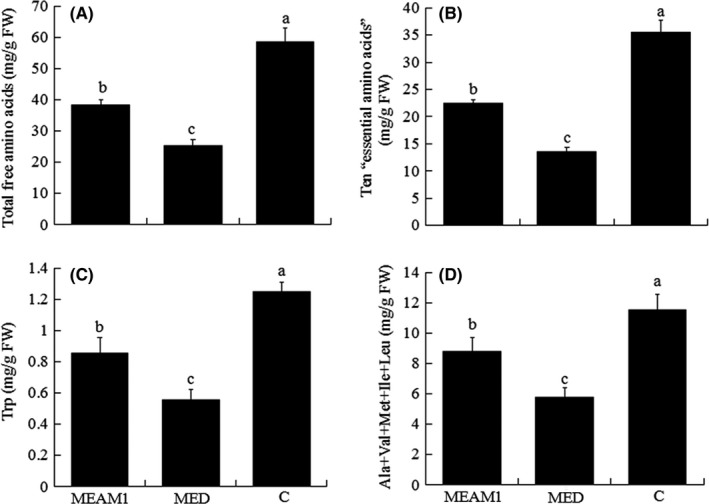
The measured concentrations of total free amino acids (A), 10 “essential amino acids” (B), Trp (C), and “Ala+Val+Met+Ile+Leu” (D) in epidermis and mesophyll tissue of cabbage plants infested with *B. tabaci* MEAM1 for 15 days, infested with MED for 15 days, or not infested (C). Values are means ± *SE* of four replicates. In each panel, means with different letters are significantly different (Tukey test: *p* < .05)

Infestation by MEAM1 or MED significantly decreased the contents of amino acids in cabbage including Trp (*F*_2, 9_ = 21.58, *p* < .0001), “Ala+Val+Met+Ile+Leu” (*F*_2, 9_ = 42.38, *p* < .0001), 10 “essential amino acids” (*F*_2, 9_ = 58.91, *p* < .0001), total amino acids (*F*_2, 9_ = 112.39, *p* < .0001), and individual amino acids (Figures 2A–D and S2).

The content of amino acids in cabbage was significantly higher with infestation by MEAM1 than MED (Figures 2A–D and S2). The amino acid content was lowest in cabbage plants infested by MED (Figure 2A–D).

### 3.3 GS content and *CYP* gene expression in cabbage plants

**Figure 3:**
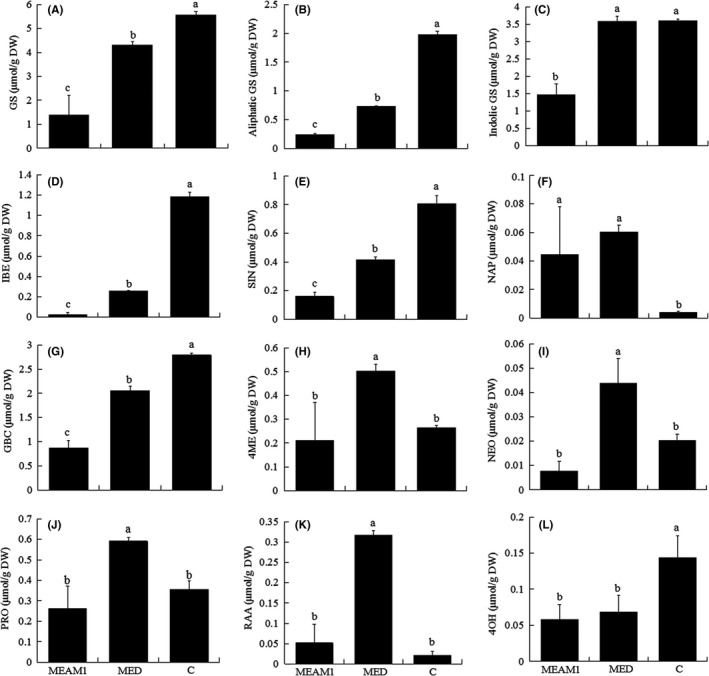
The measured concentrations of total GS (A), aliphatic GS (B), indolic GS (C), IBE (D), SIN (E), NAP (F), GBC (G), 4ME (H), NEO (I), PRO (J), RAA (K), and 4OH (L) in epidermis and mesophyll tissue of cabbage plants infested with *B. tabaci* MEAM1 for 15 days, infested with MED for 15 days, or not infested (C). Values are means ± *SE* of four replicates. In each panel, means with different letters are significantly different (Tukey test: *p* < .05)

**Figure 4:**
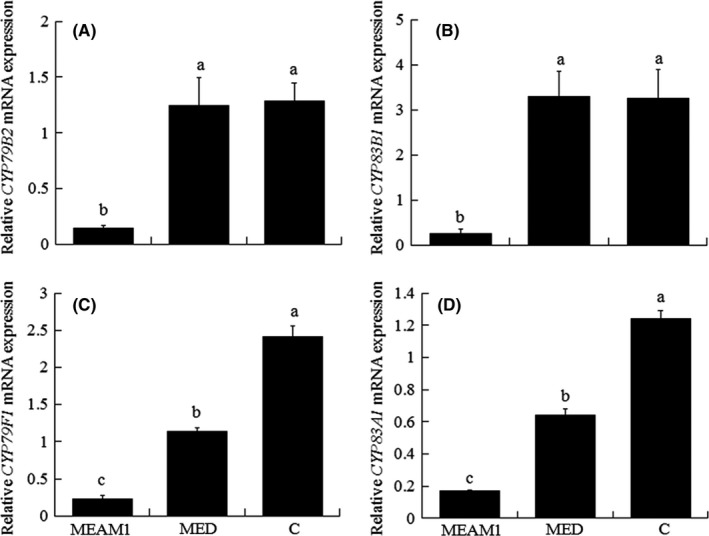
The relative expression of mRNA of *CYP79B2* (A), *CYP83B1* (B), *CYP79F1* (C), and *CYP83A1* (D) in noninfested cabbage plants (C) or cabbage plants infested with *B. tabaci* MEAM1 or MED for 15 days. Values are the means ± *SE* of four replicates. In each panel, means with different are significantly different (Tukey test: *p* < .05)

Infestation by MEAM1 or MED significantly decreased the content in cabbage of total GS, aliphatic GS, IBE (glucoiberin), SIN (sinigrin), GBC (glucobrassicin), and 4OH (4OH‐glucobrassicin) (Figure 3A,B,D,E,G,L). Infestation by MEAM1 or MED also significantly decreased the expression of *CYP79F1* and *CYP83A1* mRNA (Figure 4C,D). Infestation by MEAM1 but not by MED significantly decreased indolic GS content and *CYP79B2* and *CYP83B1* mRNA expression (Figures 3C and 4A,B). Infestation by MEAM1 or MED significantly increased the content of NAP (gluconapin) (Figure 3F). Infestation by MED but not by MEAM1 significantly increased the contents of 4ME (4ME‐glucobrassicin), NEO (neoglucobrassicin), PRO (progoitrin), and RAA (glucoraphanin) (Figure 3H–K).

The contents of the following were significantly higher in cabbage infested with MED than with MEAM1: total GS, aliphatic GS, indolic GS, IBE, SIN, GBC, 4ME, NEO, PRO, and RAA (Figure 3A–E,G–K). The expression of *CYP79B2*,* CYP83B1*,* CYP79F1*, and *CYP83A1* was significantly higher in cabbage infested with MED than with MEAM1 (Figure 4A–D).

The contents of total GS, aliphatic GS, indolic GS, IBE, SIN, GBC and expression of *CYP79B2*,* CYP83B1*,* CYP79F1*, and *CYP83A1* were lowest in cabbage plants infested by MEAM1 (Figures 3A–E,G and 4A–D). The contents of PRO, RAA, 4ME, and NEO were higher in cabbage plants infested by MED than in cabbage plants that were not infested or were infested by MEAM1 (Figure 3H–K).

## 4 DISCUSSION

Our study shows that the contents of aliphatic, indolic, and total GS were lower in cabbage infested with *B. tabaci* MEAM1 or MED than in noninfested cabbage. Previous reports showed that the quantity of GS in hosts infested with herbivorous insects may vary depending on the insect species, infestation time, plant species, and abiotic environmental factors (Falk & Gershenzon, 2007; Textor & Gershenzon, 2009). In general, GS negatively affect a wide range of herbivores, including insects, mammals, birds, mollusks, aquatic invertebrates, and nematodes (Halkier & Gershenzon, 2006; Hopkins et al., 2009). However, phloem feeders such as aphids cause little cellular damage and therefore do not generate myrosinase‐hydrolysis products, do not activate GS catabolism genes, and reduce total GS levels (Kim & Jander, 2007; Walling, 2008). Aliphatic GS content in *Arabidopsis thaliana* did not change after feeding by *Pieris rapae* (Textor & Gershenzon, 2009). High levels of aliphatic GS or indolic GS in *A. thaliana* plants, for example, decreased *B. tabaci* oviposition and development (Elbaz et al., 2012). In the current study, compared to MED, MEAM1 had higher survival, fecundity, and *r*_m_, a longer life span, and a shorter developmental time on cabbage plants. The results suggest that a reduction in GS levels in hosts benefits *B. tabaci* MEAM1 and MED but that GS levels in cabbage are reduced more by MEAM1 than by MED. In other words, the results suggest that MEAM1 is better than MED at manipulating host defenses associated with GS levels.

In the present study, we detected nine kinds of GS including three aliphatic GS (IBE, SIN, and NAP) and six indolic GS (4OH, GBC, 4ME, NEO, PRO, and RAA). Patterns and relative concentrations of these chemicals vary depending on genetic and environmental factors (Lankau & Kliebenstein, 2009; Poelman, Broekgaarden, van loon, & Dicke, 2008). A high IBE concentration was negatively associated with herbivore abundance and species richness (Kos et al., 2011; Poelman, van Dam, van Loon, Vet, & Dicke, 2009). Santolamazza‐Carbone et al. (2014) found that a high SIN content decreased the abundance of the generalist *Mamestra brassicae* (Lepidoptera, Noctuidae) and of the specialist *Plutella xylostella* (Lepidoptera, Yponomeutidae). The effects of herbivorous insects on the cytochrome P‐450 monooxygenases gene families involved in GS biosynthesis have been studied. For example, Kempema et al. (2007) demonstrated that silverleaf whitefly infestation influenced RNA levels of several genes related to sulfur and GS metabolism in *A. thaliana* (*CYP79B2* and *CYP83B1* were upregulated, while *CYP79F1*,* CYP79B3*, and *CYP83A1* were downregulated). Our study showed that MEAM1 infestation significantly decreased the expression of *CYP79B2*,* CYP83B1*,* CYP79F1*, and *CYP83A1* in cabbage plants. GS contents (including total GS, aliphatic GS, indolic GS, IBE, SIN, GBC) and the expression of related genes (*CYP79B2*,* CYP83B1*,* CYP79F1*, and *CYP83A1*) were reduced much more in cabbage infested with MEAM1 than in noninfested cabbage or cabbage infested with MED. Therefore, MEAM1 out‐performed MED on cabbage plants. These results suggest that MEAM1 regulates the GS pathway better than MED and therefore performs better than MED. In addition, infestation of cabbage by MED but not by MEAM1 significantly increased the contents of four GS components (4ME, NEO, PRO, and RAA). Other GS components in cabbage, such as IBE, SIN, GBC, and 4OH, were significantly decreased by both MEAM1 and MED. This may suggest that the differences in the levels of the four GS components may be a main reason why MEAM1 performed better than MED on cabbage plants.

Free amino acids are the precursors of several classes of secondary plant metabolites, including GS, phenolic acids, flavonoids, and others (Oliveira et al., 2008). Variation in the performance and abundance of herbivorous insects has frequently been attributed to variation in host plant quality particularly with respect to amino acid and carbohydrate levels (Cui et al., 2012; Kainulainen, Holopainen, & Holopainen, 2000). Amino acids are a limiting factor for insect growth, and there is evidence that plant amino acid composition is related to resistance to insects (Chiozza et al., 2010). The 10 “essential amino acids” have been considered the most important predictor of insect developmental time, as well as one of the most important predictors of emergence rate (Blackmer & Byr, 1999). Low levels of available amino acids in host plants adversely affect the life history traits of whiteflies and aphids (Cui et al., 2012; Girousse et al., 2005). Arg levels were positively correlated with aphid performance in a previous study (Sandström & Pettersson, 1994), and Arg was found to be an important and dominant amino acid in the current study. Numbers of adult whiteflies were positively correlated with levels of Asp, Glu, Gly, Arg, Thr, Pro, and total amino acids in cotton plants (Bi et al., 2003). In our study, although infestation by either biotype reduced the levels of amino acids in cabbage relative to levels in the noninfested control, the reduction was greater with MED than with MEAM1. The data indicated that the decreased levels of amino acids reduced the fitness of the whiteflies but that the effect was less on MEAM1 than on MED. These results suggest that MEAM1 is better than MED at managing the amino acid content of host cabbage plants for its own benefit.

As previously noted, the contents of amino acids such as Trp and “Ala+Val+Met+Ile+Leu” were higher in cabbage plants infested with MEAM1 than with MED, while the aliphatic and indolic GS contents were significantly lower in cabbage plants infested with MEAM1 than with MED. This suggests that relative to infestation with MED, infestation with MEAM1 resulted in less conversion of nutrients into those secondary metabolites that can increase host defense against herbivory.

In a previous field investigation in China, cabbage was found to be frequently infested with MEAM1 but seldom with MED (Pan et al., 2015). That same study also reported that when MEAM1 and MED were reared together on cabbage, MEAM1 competitively excluded MED after four generations in the laboratory. In the present study, MEAM1 had higher survival, higher fecundity, higher *r*_m_, a longer life span, and a shorter developmental time than MED on cabbage. Our results also showed that cabbage plants had higher levels of amino acids and lower levels of GS when infested with *B. tabaci* MEAM1 rather than MED. Our data suggest that MEAM1 performs better than MED on cabbage because MEAM1 is better able to alter cabbage nutrients and secondary metabolites to its own benefit.

Plant–insect interactions have resulted in a sophisticated arms race in which plants have developed defenses against insect attack and insects have developed various strategies to overcome or avoid these defenses (Sarmento et al., 2011; Wei et al., 2011, 2013). In the current study, we found that a biotype of *B. tabaci* MEAM1 is apparently better able to manipulate defense responses and plant nutrition to its advantage relative to MED. However, the inhibitors of the defense response in MEAM1 require investigation.

## CONFLICT OF INTEREST

None declared.

## AUTHOR CONTRIBUTIONS

Hongying Cui and Youjun Zhang conceived and designed the experiments; Hongying Cui performed the experiments; Hongying Cui and Litao Guo analyzed the data; Shaoli Wang, Wen Xie, Xiaoguo Jiao, and Qingjun Wu contributed reagents/materials/analysis tools; Hongying Cui and Youjun Zhang wrote the manuscript.

## References

[ece32921-bib-0001] Bi, J. L. , Toscano, N. C. , & Madore, M. A. (2003). Effects of urea fertilizer application on soluble protein and free amino acids content of cotton petioles in relation to silver leaf whitefly (*Bemisia argentifolii*) populations. Journal of Chemical Ecology, 29, 747–761. 12757331 10.1023/a:1022880905834

[ece32921-bib-0002] Bird, T. L. , & Kruger, K. (2006). Response of the polyphagous whitefly *Bemisia tabaci* B‐biotype (Hemiptera: Aleyrodidae) to crop diversification‐influence of multiple sensory stimuli on activity and fecundity. Bulletin of Entomological Research, 96, 15–23. 16441901 10.1079/ber2005398

[ece32921-bib-0003] Blackmer, J. L. , & Byr, D. N. (1999). Changes in amino acids in Cucumis melo in relation to life‐history traits and flight propensity of *Bemisia tabaci* . Entomologia Experimentalis et Applicata, 93, 29–40.

[ece32921-bib-0004] Chi, H. (1988). Life‐table analysis incorporating both sexes and variable development rates among individuals. Environmental Entomology, 17, 26–34.

[ece32921-bib-0005] Chi, H. , & Liu, H. (1985). Two new methods for the study of insect population ecology. Bulletin of the Institute of Zoology, Academia Sinica, 24, 225–240.

[ece32921-bib-0006] Chiel, E. , Gottlieb, Y. , Zchori‐Fein, E. , Mozes‐Daube, N. , Katzir, N. , Inbar, M. , … Ghanim, M. (2007). Biotype‐dependent secondary symbiont communities in sympatric populations of *Bemisia tabaci* . Bulletin of Entomological Research, 97, 407–413. 17645822 10.1017/S0007485307005159

[ece32921-bib-0007] Chiozza, M. V. , Oneal, M. E. , & Macintosh, G. C. (2010). Constitutive and induced differential accumulation of amino acid in leaves of susceptible and resistant soybean plants in response to the soybean aphid (Hemiptera: Aphididae). Environmental Entomology, 39(3), 856–864. 20550799 10.1603/EN09338

[ece32921-bib-0008] Cui, H. Y. , Sun, Y. C. , Su, J. W. , Li, C. Y. , & Ge, F. (2012). Reduction in the fitness of *Bemisia tabaci* fed on three previously infested tomato genotypes differing in the jasmonic acid pathway. Environmental Entomology, 41(6), 1443–1453. 23321091 10.1603/EN11264

[ece32921-bib-0009] Dardeau, F. , Body, M. A. , Berthier, A. , Miard, F. , Christides, J. P. , Feinard‐Duranceau, M. , … Salle, A. (2015). Effects of fertilisation on amino acid mobilisation by a plant‐manipulating insect. Ecological Entomology, 40(6), 814–822.

[ece32921-bib-0010] De Barro, P. J. , Liu, S. S. , Boykin, L. M. , & Dinsdale, A. (2011). *Bemisia tabaci*: a statement of species status. Annual Review of Entomology, 56, 1–19. 10.1146/annurev-ento-112408-085504 20690829

[ece32921-bib-0011] Dinsdale, A. , Cook, L. , Riginos, C. , Buckley, Y. M. , & De Barro, P. (2010). Refined global analysis of *Bemisia tabaci* (Hemiptera: Sternorrhyncha: Aleyrodoidea: Aleyrodidae) mitochondrial cytochrome oxidase 1 to identify species level genetic boundaries. Annals of the Entomological Society of America, 103, 196–208.

[ece32921-bib-0012] Elbaz, M. , Halon, E. , Malka, O. , Malitsky, S. , Blum, E. , Aharoni, A. , … Morin, S. (2012). Asymmetric adaptation to indolic and aliphatic glucosinolates in the B and Q sibling species of *Bemisia tabaci* (Hemiptera: Aleyrodidae). Molecular Ecology, 21, 4533–4546. 22849567 10.1111/j.1365-294X.2012.05713.x

[ece32921-bib-0013] Falk, K. L. , & Gershenzon, J. (2007). The desert locust, *Schistocerca gregaria*, detoxifies the glucosinolates of *Schouwia purpurea* by desulfation. Journal of Chemical Ecology, 33, 1542–1555. 17619221 10.1007/s10886-007-9331-0

[ece32921-bib-0014] Gennadius, P. (1889). Disease of tobacco plantations in Trikonia: the aleurodid of tobacco. Ellenike Georgia, 5, 1–3.

[ece32921-bib-0015] Girousse, C. , Moulia, B. , Silk, W. , & Bonnemain, J. L. (2005). Aphid infestation causes different changes in carbon and nitrogen allocation in alfalfa stems as well as different inhibitions of longitudinal and radial expansion. Plant Physiology, 137, 1474–1484. 15778456 10.1104/pp.104.057430 PMC1088336

[ece32921-bib-0016] Gols, R. , Bukovinszky, T. , van Dam, N. M. , Dicke, M. , Bullock, J. M. , & Harvey, J. A. (2008). Performance of generalist and specialist herbivores and their endoparasitoids differs on cultivated and wild *Brassica* populations. Journal of Chemical Ecology, 34, 132–143. 18231835 10.1007/s10886-008-9429-z PMC2239250

[ece32921-bib-0017] Gols, R. , Wagenaar, R. , Bukovinszky, T. , van Dam, N. M. , Dicke, M. , Bullock, J. M. , … Harvey, J. A. (2008). Genetic variation in defense chemistry in wild cabbages affects herbivores and their endoparasitoids. Ecology, 89, 1616–1626. 18589526 10.1890/07-0873.1

[ece32921-bib-0018] Gomes, M. H. , & Rosa, E. (2001). Free amino acid composition in primary and secondary inflorescences of 11 broccoli (*Brassica oleracea* var *italica*) cultivars and its variation between growing seasons. Journal of the Science of Food and Agriculture, 81, 295–299.

[ece32921-bib-0019] Guo, Z. J. , Kang, S. , Chen, D. F. , Wu, Q. J. , Wang, S. L. , Xie, W. , … Zhang, Y. J. (2015). The MAPK signaling pathway alters expression of midgut receptor genes and confers resistance to *Bacillus thuringiensis* Cry1Ac toxin in diamondback moth. PLoS Genetics, 11(4), e1005124. 25875245 10.1371/journal.pgen.1005124 PMC4395465

[ece32921-bib-0020] Halkier, B. A. , & Gershenzon, J. (2006). Biology and biochemistry of glucosinolates. Annual Review of Plant Biology, 57, 303–333. 10.1146/annurev.arplant.57.032905.105228 16669764

[ece32921-bib-0021] Heaney, R. K. , Spinks, E. A. , Hanley, A. B. , & Fenwick, G. R. (1986). Analysis of glucosinolates in rapeseed, Technical Bulletin. Norwich, UK: AFRC Food Research Institute.

[ece32921-bib-0022] Hopkins, R. J. , van Dam, N. , & van loon, J. J. A. (2009). Role of glucosinolates in insect‐plant relationships and multitrophic interactions. Annual Review of Entomology, 54, 57–83. 10.1146/annurev.ento.54.110807.090623 18811249

[ece32921-bib-0023] Iida, H. , Kitamura, T. , & Honda, K. (2009). Comparison of egg‐hatching rate, survival rate and development time of the immature stage between B‐ and Q‐biotypes of *Bemisia tabaci* (Gennadius) (Homoptera: Aleyrodidae) on various agricultural crops. Applied Entomology and Zoology, 44, 267–273.

[ece32921-bib-0024] Jiao, X. G. , Xie, W. , Wang, S. L. , Wu, Q. J. , Pan, H. P. , Liu, B. M. , … Zhang, Y. J. (2013). Differences in host selection and performance between B and Q putative species of *Bemisia tabaci* on three host plants. Entomologia Experimentalis et Applicata, 147, 1–8.

[ece32921-bib-0025] Jones, D. R. (2003). Plant viruses transmitted by whiteflies. European Journal of Plant Pathology, 109, 195–219.

[ece32921-bib-0026] Kainulainen, P. , Holopainen, J. , & Holopainen, T. (2000). Combined effects of ozone and nitrogen on secondary compounds, amino acids, and aphid performance in Scots pine seedlings. Journal of Environmental Quality, 29, 334–342.

[ece32921-bib-0027] Karley, A. J. , Douglas, A. E. , & Parker, W. E. (2002). Amino acid composition and nutritional quality of potato leaf phloem sap for aphids. Journal of Experimental Botany, 205, 3009–3018. 10.1242/jeb.205.19.3009 12200404

[ece32921-bib-0028] Kempema, L. A. , Cui, X. P. , Holzer, F. M. , & Walling, L. L. (2007). Arabidopsis transcriptome changes in response to phloem‐feeding silverleaf whitefly nymphs. similarities and distinctions in responses to aphids. Plant Physiology, 143, 849–865. 17189325 10.1104/pp.106.090662 PMC1803730

[ece32921-bib-0029] Kim, J. H. , & Jander, G. (2007). *Myzus persicae* (green peach aphid) feeding on Arabidopsis induces the formation of a deterrent indole glucosinolate. The Plant Journal, 49, 1008–1019. 17257166 10.1111/j.1365-313X.2006.03019.x

[ece32921-bib-0030] Kos, M. , Broekgaarden, C. , Kabouw, P. , Oude Lenferink, K. , Poelman, E. H. , Vet, L. E. M. , … van loon, J. J. A. (2011). Relative importance of plant‐mediated bottom‐up and top‐down forces on herbivore abundance on *Brassica oleracea* . Functional Ecology, 25, 1113–1124.

[ece32921-bib-0031] Lankau, R. A. , & Kliebenstein, D. J. (2009). Competition, herbivory and genetics interact to determine the accumulation and fitness consequences of a defence metabolite. Journal of Ecology, 97, 78–88.

[ece32921-bib-0032] Leroy, P. D. , Wathelet, B. , Sabri, A. , Francis, F. , Verheggen, F. J. , Capella, Q. , … Haubrug, E. (2011). Aphid‐host plant interactions: does aphid honeydew exactly reflect the host plant amino acid composition? Arthropod‐Plant Interactions, 5(3), 193–199.

[ece32921-bib-0033] Livak, K. J. , & Schmittgen, T. D. (2001). Analysis of relative gene expression data using real‐time quantitative PCR and the 2^‐△△Ct^ method. Methods, 25, 402–408. 11846609 10.1006/meth.2001.1262

[ece32921-bib-0034] Louis, J. , Singh, V. , & Shah, J. (2012). Arabidopsis thaliana‐Aphid Interaction. The Arabidopsis Book, 10, e0159. Published online. 22666177 10.1199/tab.0159 PMC3365623

[ece32921-bib-0035] Markovich, O. , Kafle, D. , Elbaz, M. , Malitsky, S. , Aharoni, A. , Schwarzkopf, A. , … Morin, S. (2013). *Arabidopsis thaliana* plants with different levels of aliphatic and indolyl‐glucosinolates affect host selection and performance of *Bemisia tabaci* . Journal of Chemical Ecology, 39, 1361–1372. 24190022 10.1007/s10886-013-0358-0

[ece32921-bib-0036] Martinez‐Villaluenga, C. , Peñas, E. , Frias, J. , Ciska, E. , Honke, J. , Piskula, M. K. , … Vidal‐Valverde, C. (2009). Influence of fermentation conditions on glucosinolates, ascorbigen, and ascorbic acid content in white cabbage (*Brassica oleracea* var. *capitata*cv. Taler) cultivated in different seasons. Journal of Food Science, 74(1), 62–67. 10.1111/j.1750-3841.2008.01017.x 19200088

[ece32921-bib-0037] McKenzie, C. L. , Hodges, G. , Osborne, L. S. , Byrne, F. J. , & Shatters, R. G. (2009). Distribution of *Bemisia tabaci* (Hemiptera: Aleyrodidae) biotypes in Florida–investigating the Q invasion. Journal of Economic Entomology, 102, 670–676. 19449648 10.1603/029.102.0227

[ece32921-bib-0038] Mewis, I. , Tokuhisa, J. G. , Schultz, J. C. , Appel, H. M. , Ulrichs, C. , & Gershenzon, J. (2006). Gene expression and glucosinolate accumulation in *Arabidopsis thalianain* response to generalist and specialist herbivores of different feeding guilds and the role of defense signaling pathways. Phytochemistry, 67, 2450–2462. 17049571 10.1016/j.phytochem.2006.09.004

[ece32921-bib-0039] Müller, C. , & Sieling, N. (2006). Effects of glucosinolate and myrosinase levels in *Brassica juncea* on a glucosinolate‐sequestering herbivore‐and *vice versa* . Chemoecology, 16, 191–201.

[ece32921-bib-0040] Newton, E. L. , Bullock, J. M. , & Hodgson, D. J. (2009). Glucosinolate polymorphism in wild cabbage (*Brassica oleracea*) influences the structure of herbivore communities. Oecologia, 160, 63–76. 19214588 10.1007/s00442-009-1281-5

[ece32921-bib-0041] Ohgushi, T. (2005). Indirect interaction webs: herbivore‐induced effects through trait change in plants. Annual Review of Ecology, Evolution, and Systematics, 36, 81–105.

[ece32921-bib-0042] Oliveira, A. P. , Pereira, D. M. , Andrade, P. B. , Valentao, P. , Sousa, C. , Pereira, J. , … Silva, A. M. (2008). Free amino acids of tronchuda cabbage (*Brassica oleracea* L. Var. *costata* DC): influence of leaf position (internal or external) and collection time. Journal of Agricultural and Food Chemistry, 56, 5216–5221. 18553888 10.1021/jf800563w

[ece32921-bib-0043] Pan, H. P. , Chu, D. , Liu, B. M. , Shi, X. B. , Guo, L. T. , Xie, W. , … Zhang, Y. J. (2013). Differential effects of an exotic plant virus on its two closely related vectors. Scientific Reports, 3, 2230. 23864010 10.1038/srep02230 PMC3714654

[ece32921-bib-0044] Pan, H. P. , Preisser, E. , Chu, D. , Wang, S. L. , Wu, Q. J. , Carriere, Y. , … Zhang, Y. J. (2015). Insecticides promote viral outbreaks by altering herbivore competition. Ecological Applications, 25(6), 1585–1595. 26552266 10.1890/14-0752.1

[ece32921-bib-0045] Petersen, M. K. , & Sandstrom, J. P. (2001). Outcome of indirect competition between two aphid species mediated by responses in their common host plant. Functional Ecology, 15, 525–534.

[ece32921-bib-0046] Pieterse, C. M. J. , & Dicke, M. (2007). Plant interactions with microbes and insects: from molecular mechanisms to ecology. Trends in Plant Science, 12(12), 1365–1380. 10.1016/j.tplants.2007.09.004 17997347

[ece32921-bib-0047] Poelman, E. H. , Broekgaarden, C. , van loon, J. J. A. , & Dicke, M. (2008). Early season herbivore differentially affects plant defence responses to subsequently colonizing herbivores and their abundance in the field. Molecular Ecology, 17, 3352–3365. 18565114 10.1111/j.1365-294X.2008.03838.x

[ece32921-bib-0048] Poelman, E. H. , van Dam, N. , van Loon, J. J. A. , Vet, L. E. M. , & Dicke, M. (2009). Chemical diversity in *Brassica oleracea* affects biodiversity of insect herbivores. Ecology, 90, 1863–1877. 19694135 10.1890/08-0977.1

[ece32921-bib-0049] Renwick, J. A. A. , & Lopez, K. (1999). Experience‐based food consumption by larvae of *Pieris rapae*: Addiction to glucosinolates? Entomologia Experimentalis et Applicata, 91, 51–58.

[ece32921-bib-0050] Sandström, J. , & Pettersson, J. (1994). Amino acid composition of phloem sap and the relation to intraspecific variation in pea aphid (*Acyrthosiphon pisum*) performance. Journal of Insect Physiology, 40(11), 947–955.

[ece32921-bib-0051] Santolamazza‐Carbone, S. , Velasco, P. , Soengas, P. , & Cartea, M. E. (2014). Bottom‐up and top down herbivore regulation mediated by glucosinolates in *Brassica oleracea* var. Acephala. Oecologia, 174, 893–907. 24352843 10.1007/s00442-013-2817-2

[ece32921-bib-0052] Sarmento, R. A. , Lemos, F. , Bleeker, P. M. , Schuurink, R. C. , Pallini, A. , Oliveira, A. M. G. , … Janssen, A. (2011). A herbivore that manipulates plant defence. Ecology Letters, 14, 229–236. 21299823 10.1111/j.1461-0248.2010.01575.x PMC3084520

[ece32921-bib-0053] Su, Q. , Oliver, K. M. , Xie, W. , Wu, Q. J. , Wang, S. L. , & Zhang, Y. J. (2015). The whitefly‐associated facultative symbiont Hamiltonella defense suppress induced plant defenses in tomato. Functional Ecology, 29(8), 1007–1018.

[ece32921-bib-0054] Su, Q. , Perisser, E. L. , Zhou, X. M. , Xie, W. , Liu, B. M. , Wang, S. L. , … Zhang, Y. J. (2015). Manipulation of host quality and defense by a plant virus improves performance of whitefly vectors. Journal of Economic Entomology, 108(1), 11–19. 26470098 10.1093/jee/tou012

[ece32921-bib-0055] Textor, S. , & Gershenzon, J. (2009). Herbivore induction of the glucosinolate‐myrosinase defense system: major trends, biochemical bases and ecological significance. Phytochemistry Reviews, 8, 149–170.

[ece32921-bib-0056] Thompson, G. A. , & Goggin, F. L. (2006). Transcriptomics and functional genomics of plant defence induction by phloem‐feeding insects. Journal of Experimental Botany, 57, 755–766. 16495409 10.1093/jxb/erj135

[ece32921-bib-0057] Walling, L. L. (2008). Avoiding effective defenses: strategies employed by phloem‐feeding insects. Plant Physiology, 146, 859–886. 18316641 10.1104/pp.107.113142 PMC2259051

[ece32921-bib-0058] Wei, J. N. , Wang, L. Z. , Zhao, J. H. , Li, C. Y. , Ge, F. , & Kang, L. (2011). Ecological trade‐offs between jasmonic acid‐dependent direct and indirect plant defenses in tritrophic interactions. New Phytologist, 189, 557–567. 21039561 10.1111/j.1469-8137.2010.03491.x PMC3039750

[ece32921-bib-0059] Wei, J. N. , Yan, L. , Ren, Q. , Li, C. Y. , Ge, F. , & Kang, L. (2013). Antagonism between herbivore‐induced plant volatiles and trichomes affects tritrophic interactions. Plant, Cell and Environment, 36, 315–327. 10.1111/j.1365-3040.2012.02575.x 22789006

[ece32921-bib-0060] Wilkinson, T. L. , & Douglas, A. E. (2003). Phloem amino acids and the host plant range of the polyphagous aphid, *Aphis fabae* . Entomologia Experimentalis et Applicata, 106, 103–113.

[ece32921-bib-0061] Will, T. , & van Bel, A. J. E. (2008). Induction as well as suppression: How aphid saliva may exert opposite effects on plant defense. Plant Signaling & Behavior, 3(6), 427–430. 19704587 10.4161/psb.3.6.5473 PMC2634323

[ece32921-bib-0062] Zhang, L. P. , Zhang, Y. J. , Zhang, W. J. , Wu, Q. J. , Xu, B. Y. , & Chu, D. (2005). Analysis of genetic diversity among different geographical populations and determination of biotypes of *Bemisia tabaci* in China. Journal of Applied Entomology, 129, 121–128.

