# Supplementary material for: The ability to manipulate plant glucosinolates and nutrients explains the better performance of Bemisia tabaci Middle East‐Asia Minor 1 than Mediterranean on cabbage plants
Source: Ecol Evol. 2017 Jun 30;7(16):6141–50. doi: 10.1002/ece3.2921 (PMC5574797; doi:10.1002/ece3.2921)
Supplement: Supplementary file 1 [file ECE3-7-6141-s001.doc]

Supporting Information

**Table S1.** The average number of egg, larva and adult (mean ± SE) per cabbage plant oviposited by adult whitefly MEAM1 and MED after 9 days, 12days and 15days of development respectively.

|  | Biotypes | Eggs | Larva | Adults |
| --- | --- | --- | --- | --- |
| 15days | MEAM1 | 4230±170.51a | 1545±84.67a | 450a |
|  | MED | 4190±215.66a | 1481±119.62a | 450a |
| 12days | MEAM1 | 4494±110.40a | 1214±99.29a | 450a |
|  | MED | 4405±175.39a | 1167±105.62a | 450a |
| 9days | MEAM1 | 4674±108.37a | 1011±97.59a | 450a |
|  | MED | 4565±165.35a | 965±101.06a | 450a |

**Table S2.** Primer sequences used for real-time quantitative PCR.

| Gene ID | Gene name | Primer sequence（5’-3’） | Fragment length (bp) | Function |
| --- | --- | --- | --- | --- |
| Bol018585 | *CYP79B2* | F: CTTACGCGCAAAACGTCCTC  R: ATGAAGCCACCTGTGTCTCG | 163 | indolic GS related |
| Bol033477 | *CYP83B1* | F: TGAGGAATGTGGTCGGTGAC  R: GACTGGTTCGAGACGGAGTG | 147 | indolic GS related |
| Bol038222 | *CYP79F1* | F: TCGCATGTTTCAACTTCGCC  R: GCCTCCGATGGTTCTCATGT | 155 | aliphatic GS related |
| Bol040365 | *CYP83A1* | F: GCAATGAACCACTACACACCG  R: TATCGACAGGTTCGGCTCTC | 151 | aliphatic GS related |
| Bol030974 | *Actin* | F: TCCCAGGGCTGTTTTCCCTA  R: TCCCAGTTGCTCACAACACC | 113 | Housekeeping gene |
| Bol010398 | *GADPH* | F: ACATCATTCCCAGCAGCAC  R: GCTTGCCCTCAGATTCCT | 102 | Housekeeping gene |

**Table S3.** Results of the ANOVA performed to test the effects of whitefly biotype and generation on the *B. tabaci* population parameters.

| Population parameters | Biotype | | Generation | | Biotype×Generation | |
| --- | --- | --- | --- | --- | --- | --- |
|  | F | *P* | F | *P* | F | *P* |
| r_m_^a^ | 5600.45 | < 0.0001 | 26.48 | < 0.0001 | 44.52 | < 0.0001 |
| Developmental time | 1262.16 | < 0.0001 | 4.47 | 0.004 | 1.78 | 0.149 |
| Fecundity | 455.77 | < 0.0001 | 6.44 | < 0.0001 | 9.57 | < 0.0001 |
| Lifespan | 119.68 | < 0.0001 | 0.96 | 0.412 | 1.07 | 0.363 |
| Survival | 593.21 | < 0.0001 | 0.13 | 0.943 | 7.24 | < 0.0001 |

^a^ intrinsic rate of increase

**Figure S1.**

**
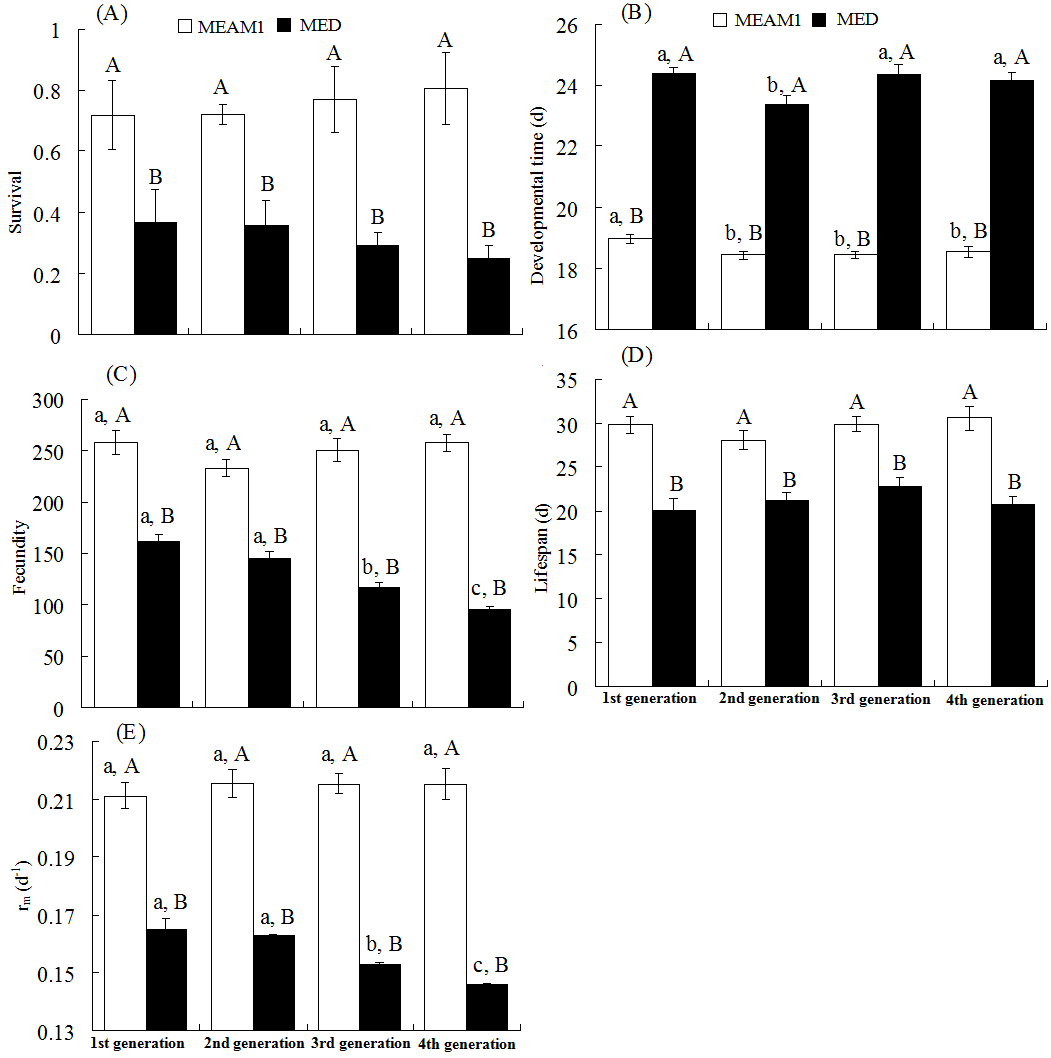
Figure S1.** Survival (A), developmental time (from egg to adult) (B), fecundity (C), lifespan (D), and intrinsic rate of increase (r_m_) (E) of *B. tabaci* MEAM1 and MED for generations 1 to 4 on cabbage. Values are means ±SE across all individuals within a treatment. Within a biotype and a panel, different lowercase letters indicate a significant difference among generations. Within a generation and a panel, different uppercase letters indicate significant differences between biotypes (Tukey test: *P*< 0.05).

**Figure S2.**

**
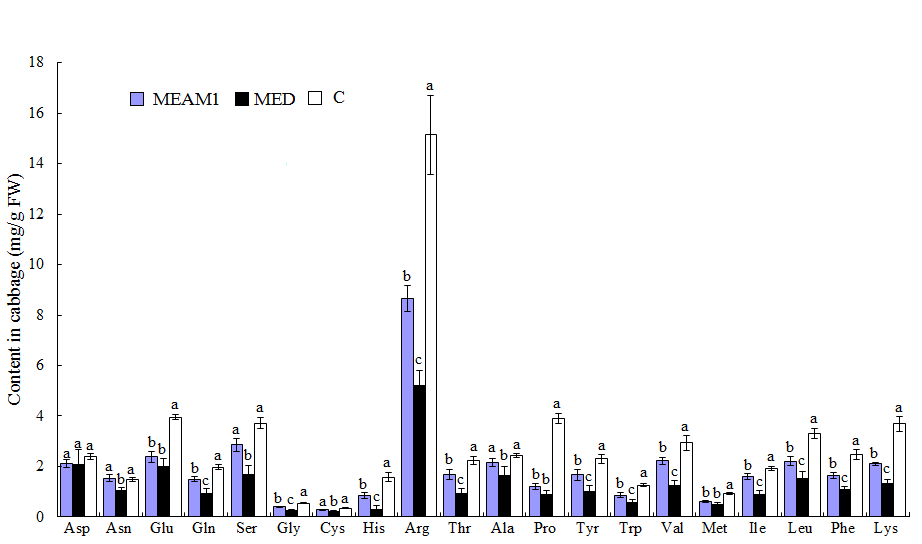
**

**Figure S2.** The measured concentrations of free amino acids in epidermis and mesophyll tissue of cabbage plants infested with *B. tabaci* MEAM1 for 15 days, infested with MED for 15 days, or not infested (C). Value are means ±SE of four replicates. For each amino acid, means with different letters are significantly different (Tukey test: *P*< 0.05).
